# Clinical Characteristics of Ischemic Stroke Patients <50 Years Old at a University Hospital: A Retrospective Descriptive Study

**DOI:** 10.7759/cureus.43752

**Published:** 2023-08-19

**Authors:** Abdullah Alkutbi, Saleh Binmahfooz, Rawan H AlSaidlani, Rasana B Albeirouti, Omar Kamal, Hassan Alalawi, Mohammed N Aljehani, Mohsin Khared, Omar A Ayoub

**Affiliations:** 1 Department of Internal Medicine, King Abdulaziz University Faculty of Medicine, Jeddah, SAU; 2 Department of Internal Medicine, King Abdulaziz University Hospital, Jeddah, SAU; 3 Deparmtent of Internal Medicine, King Abdulaziz University Faculty of Medicine, Jeddah, SAU

**Keywords:** hypertension, diabetes mellitus, young adult, risk factors, acute ischemic stroke

## Abstract

Background

Stroke is a leading cause of mortality and disability around the world. It is responsible for 10% of all fatalities and about 5% of all disabilities. Risk factors include age, hypertension (HTN), dyslipidemia, and atrial fibrillation. The incidence of acute ischemic stroke (AIS) is increasing among young adults compared to older ones. It has a direct impact on their quality of life and working activities while also burdening the healthcare system.

Aim

The aim of this study is to investigate the possible risk factors for ischemic stroke in patients who are under 50 years old.

Methods

This is a single-center retrospective record review of patients with ischemic stroke from 2010 to 2022. Eighty patients who had an ischemic stroke at an age below 50 were included in the analysis. Patients above or equal to 50 years old who had ischemic stroke and all patients with hemorrhagic stroke were excluded. Baseline characteristics, length of hospitalization, and in-hospital mortality were compared with different comorbidities.

Results

The mean age was 36.65 among males and females who had an ischemic stroke. 56.8% of them were non-Saudi, while 43.2% were Saudis. Diabetes, hypertension, and dyslipidemia were among the most frequent comorbidities among patients who had ischemic stroke, with a percentage of 82.7%. Other comorbidities, such as autoimmune disease, thrombophilia, and heart failure, were present.

Conclusion

There are different comorbidities found in patients who have had an ischemic stroke and are under 50 years old. However, diabetes and hypertension remain the most common risk factors.

## Introduction

Stroke is the leading cause of mortality and disability worldwide. It accounts for 10% of all fatalities and approximately 5% of all disabilities [1]. Stroke is predicted to be the main cause of the loss of healthy life years due to the aging of the global population [2]. Risk factors for stroke include age, hypertension (HTN), dyslipidemia, and atrial fibrillation. In Saudi Arabia, the incidence rate of all strokes varies from 175.8 to 196.2 per 100,000 individuals, whereas the rate of intracerebral hemorrhage varies from 39.7 to 48.6 per 100,000 individuals. In addition, the incidence rates for acute ischemic stroke (AIS) range from 131.0 to 151.5 [3]. Another study in China showed that ischemic stroke accounts for 79.1% of all stroke types [4-6]. There are significant numbers of ischemic strokes that occur in young patients [7], who have distinct risk factors compared to older patients [8]. Possible risk factors were addressed in a large study done in Europe in 2012 that included 3944 patients with a median age of 43 years and concluded that the most common risk factors among young individuals were smoking (49%), dyslipidemia (46%), and HTN (36%) [9]. In contrast, a study in Japan in 2022 revealed that dyslipidemia, HTN, and diabetes mellitus (DM) were less frequent among young patients experiencing ischemic stroke. Moreover, smoking, alcohol consumption, and obesity were more frequent risk factors for young individuals [10]. AIS among young adults has a huge impact on the quality of life, such as the loss of the ability to work and a significant burden on the healthcare system [11-13]. Therefore, effective primary and secondary prevention measures for young individuals will have a huge impact on their quality of life [9]. A small number of studies that focus on young adults have been published in the literature, and most of them were single-center cohorts [9]. Therefore, the insufficient number of studies addressing young populations with AIS and related risk factors makes it an important area of research. This study aimed to investigate the possible risk factors for AIS in patients aged <50 years at King Abdulaziz University Hospital, Jeddah, Saudi Arabia.

## Materials and methods

Sample description

In this study, we aimed to identify risk factors in young patients diagnosed with AIS at King Abdulaziz University Hospital, a tertiary care center, teaching hospital, and referral hospital in the city of Jeddah, Saudi Arabia.

We conducted a retrospective cross-sectional study wherein we reviewed the medical records of inpatients, outpatients, intensive care units, and emergency medicine departments, searching for patients with a clinical diagnosis of ischemic stroke, either first-time AIS or recurrent AIS, between 2010 and 2022. Eighty-one patients under 50 years old with ischemic stroke were included, excluding those with hemorrhagic stroke or over 49 years old. Most were of Middle Eastern descent.

Risk factors

We investigated the baseline characteristics of the patients, including demographics, history of smoking, and comorbidities. The definitions of risk factors were as follows: diabetes mellitus (fasting blood glucose concentration ≥7.0 mmol/L, positive 75-g oral glucose tolerance test, or glycated hemoglobin A1c concentration of 6.5%), hypertension (systolic blood pressure of ≥140 mmHg or diastolic blood pressure of ≥90 mmHg), dyslipidemia (elevated total or low-density lipoprotein [LDL] cholesterol levels, or low levels of high-density lipoprotein [HDL] cholesterol), smoking (current or former cigarette smoker), thrombophilia (a blood disorder that makes the blood in veins and arteries more likely to clot known as "hypercoagulable state"), autoimmune disease (a disease of which the body immune system responds to a functioning body part).

Ethics statement

The Research Ethics Committee NCBE registration (No. HA-02-J-008) at King Abdulaziz University reviewed and approved our research proposal with ethical approval (reference No. 556-22). The requirement for consent was waived due to the retrospective nature of the study.

Data collection

Clinical data were obtained from the hospital record, which included demographic data, smoking history, length of hospitalization in the setting of AIS, in-hospital death, and comorbidities that included multiple chronic diseases such as diabetes mellitus (DM), HTN, dyslipidemia, and acute coronary syndrome.

Data entry and statistical analysis

Data entry was performed using Google Sheets and then transferred to Excel version 16.70. We analyzed the data using IBM SPSS Statistics for Windows version 21. Means ± standard deviations were calculated for age, length of hospitalization, and scaled data. Additionally, we calculated the frequencies and percentages for the categorical data. We used the Chi-square test to determine the relationship between comorbidities and in-hospital mortality. The t-test was used to compare the length of hospitalization and comorbidities. Statistical significance was considered when the P-value was equal to or less than 0.05.

## Results

Baseline characteristics

Eighty-one patients were included in the study. A total of 52 (64.2%) patients were female, and 29 (35.8%) were male. The mean age of the patients was 36.65 ± 12.47 years. Regarding nationality, 46 (56.8%) patients were non-Saudi, and 35 (43.2%) were Saudi. The most common comorbidities were HTN and DM. Table 1 summarizes baseline patient characteristics.

**Table 1:** Length of hospitalization for patients with different comorbidities

Comorbidity	No	Yes	P-value
Diabetes mellitus	7.94 ± 13.36	15.67 ± 23.51	0.063
Hypertension	8.32 ± 13.40	14.68 ± 23.43	0.124
Dyslipidemia	11.19 ± 18.68	6.67 ± 9.42	0.416
Coronary artery disease	9.12 ± 13.05	28 ± 45.44	0.356
Acute coronary syndrome	9.21 ± 12.99	30.40 ± 50.38	0.401
Congenital heart disease	10.43 ± 17.95	12.25 ± 11.79	0.842
Autoimmune disease	10.78 ± 18.01	5.50 ± 6.80	0.236
Thrombophilia	11.71 ± 18.69	2.91 ± 2.77	0.125
Cerebral venous thrombosis	10.97 ± 18.07	3.60 ± 6.50	0.369
Seizure disorder	10.91 ± 18.00	3.00 ± 2.94	0.385
Heart failure	10.84 ± 18.42	7.63 ± 7.50	0.628
Chronic kidney disease	10.76 ± 17.93	4.33 ± 3.51	0.539
Hyperthyroidism	10.63 ± 17.23	6.00 ± 8.49	0.716
Hypothyroidism	10.76 ± 17.93	4.33 ± 4.51	0.539

Length of hospitalization

**Figure 1:**
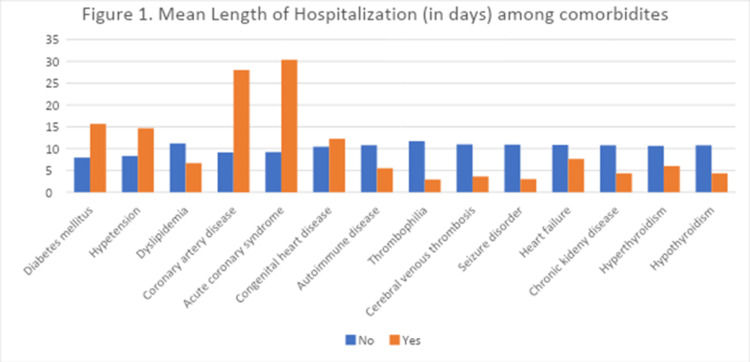
Mean length of hospitalization (in days) among comorbidities

The mean length of hospitalization for male patients was 14.55 ± 26.89 days compared to 8.27 ± 8.77 days for female patients; the difference was not statistically significant (P=0.231). The mean length of hospitalization for patients with no prior history of smoking was 10.75 ± 18.17 days compared to 7.00 ± 4.18 days for patients with a positive history of smoking, without a significant difference (P=0.648). Table 2 and Figure 1 demonstrate a comparison of the mean length of hospitalization between patients with different comorbidities.

 

**Table 2:** In-hospital mortality for patients with different comorbidities

Comorbidity	No in-hospital mortality	In-hospital mortality	P-value
Diabetes mellitus	20	7	0.363
Hypertension	23	5	1.000
Dyslipidemia	12	0	0.110
Coronary artery disease	5	1	1.000
Acute coronary syndrome	4	1	1.000
Congenital heart disease	4	0	1.000
Autoimmune disease	3	1	0.567
Thrombophilia	11	0	0.203
Cerebral venous thrombosis	5	0	0.578
Seizure disorder	4	0	1.000
Heart failure	6	2	0.637
Chronic kidney disease	2	1	0.464
Hyperthyroidism	1	1	0.338
Hypothyroidism	2	1	0.464

In-hospital mortality

During their hospitalization, 18.5% of patients experienced in-hospital mortality, of whom six (7.4%) were males and nine (11.11%) were females. Among patients with a history of smoking, two (2.47%) experienced in-hospital mortality, whereas three (3.70%) did not. All of which showed no statistical significance. Table 3 shows a comparison between patients with different comorbidities regarding in-hospital mortality.

**Table 3:** Baseline characteristics *Length of hospitalization in days

Characteristics	Results
Age (mean ± SD)	36.65 ± 12.47
Gender (n, %)	
Male	29 (35.8%)
Female	52 (64.2%)
Nationality (n, %)	
Saudi	35 (43.2%)
Non-Saudi	46 (56.8%)
Length of hospitalization* (mean ± SD)	10.52 ± 17.65
History of smoking (n, %)	5 (6.2%)
Comorbidities (n, %)	
Diabetes mellitus	27 (33.3%)
Hypertension	28 (34.6%)
Dyslipidemia	12 (14.8%)
Coronary artery disease	6 (7.4%)
Acute coronary syndrome	5 (6.2%)
Atrial fibrillation	1 (1.2%)
Congenital heart disease	4 (4.9%)
Heart failure	8 (9.9%)
Autoimmune disease	4 (4.9%)
Thrombophilia	11 (13.6%)
Sickle cell anemia	1 (1.2%)
Cerebral venous thrombosis	5 (6.2%)
Seizure disorder	4 (4.9%)
Malignancy	1 (1.2%)
Chronic kidney disease	3 (3.7%)
Hyperthyroidism	2 (2.5%)
Hypothyroidism	3 (3.7%)
Previous history of stroke or TIA (n, %)	7 (8.6%)

## Discussion

Baseline characteristics

The average age of the participants in our study was 36.65 years. This was slightly higher than the mean age of 30.1 years reported in a local study by Alamei et al. [14] and lower than the mean age of 37 years reported in a study of Chinese patients [15]. This indicates that younger people are more likely to experience strokes. Additionally, they may be more likely to engage in risky behaviors, such as smoking, that can lead to strokes.

We found that 64.2% of the patients were female. This was comparable to the findings of a regional study in which 66.5% of study participants were female [14]. A study conducted in Riyadh, on the other hand, reported a majority of male patients (68.2%) [16]. According to these data, young women have a higher risk of stroke than men, which can be attributed to a combination of biological and lifestyle factors. For example, women are more likely to have HTN, DM, and obesity, all of which increase the risk of stroke.

According to our findings, HTN and DM were the most common comorbidities among the patients. This finding is consistent with those of previous studies published in the literature [15,17]. Hypothetically, HTN and DM come with other metabolic disorders such as obesity and hypercholesterolemia, which further increase the risk of stroke [7]. HTN and DM are among the modifiable risk factors for stroke; therefore, it is crucial for individuals with HTN and DM to prioritize the management of their blood pressure and glucose levels to minimize the risk of stroke and its detrimental consequences [7,11].

Length of hospitalization

Our study included 81 patients with ischemic stroke and investigated their length of hospital stay and the factors that contributed to it. The results showed no positive correlation between the length of hospitalization and comorbidities. The most common comorbidity in our population was DM, followed by HTN; they were associated with an average of 20 days of hospitalization. However, there was no statistically significant correlation between variables and the duration of hospitalization. Similarly, in previous studies, DM and HTN were not associated with extended hospital stays of more than seven days, which might indicate that comorbidities are not contributing factors to hospitalization duration [18,19]. Since comorbidities have not been linked to a longer hospital stay in our study, perhaps other factors like the type and severity of stroke would play a role in the length of hospitalization. The study by Hakim et al. showed that shorter hospital stays were linked to stroke types that did not involve the total anterior circulation [20]. Furthermore, a study in Peru stated that a longer length of stay was associated with hemorrhagic strokes rather than ischemic or other types [21]. We believe that proper control of comorbidities during hospitalization is crucial. Still, it is also very important to investigate and control the other factors that prolong a patient’s stay in the hospital.

In-hospital mortality

This study revealed factors responsible for in-hospital mortality in selected cases. We observed that 15 (18.5%) patients died during their hospital stay, of whom six (7.4%) were males and nine (11.11%) were females. Five (6.17%) patients had a positive history of smoking, of whom two (2.47%) died and three (3.70%) did not. Possible causes of mortality were analysed, and two conditions were found to have a significant impact on mortality: DM and HTN. Seven of the deceased patients had DM, and five had HTN. Among the other patients who died, one had CAD and one had acute coronary syndrome. Three patients had end-organ diseases, two had thyroid gland disorders, and one had an autoimmune disease. A 2018 meta-analysis that reviewed 66 eligible articles revealed that hyperglycemia and DM were correlated with an overall poor stroke outcome, including higher mortality and readmission rates, longer hospital stays, and recurrences [22]. We hypothesize that mortality in stroke patients with long-standing conditions similar to DM and HTN may be due to the overall exhaustion of patients suffering from comorbidities. This might eventually lead to an overall decrease in patient tolerance and, therefore, an increased risk of mortality.

In the present study, we sought to evaluate different risk factors associated with AIS in the population younger than 50 years; however, there was a limitation of missing data in our hospital; therefore, a multicenter study is needed to access a larger population with more diverse data. Furthermore, appropriate documentation of comorbid conditions and risks would greatly aid subsequent research.

## Conclusions

Based on our retrospective study, it was found that patients under the age of 50 who had AIS were often linked to various comorbidities. However, the most common risk factors found in the study were DM and HTN. To obtain a more conclusive result, further studies should be conducted in larger institutions and teaching hospitals.
